# Meta‐analysis of high‐intensity interval training and alternative modalities for enhancing aerobic and anaerobic endurance in young athletes

**DOI:** 10.14814/phy2.70598

**Published:** 2025-10-05

**Authors:** Manuel Matzka, Mascha Lenk, Benedikt Meixner, Billy Sperlich

**Affiliations:** ^1^ Department of Sport Science Julius‐Maximilians‐Universität Würzburg, Integrative and Experimental Exercise Science & Training Würzburg Germany; ^2^ Department for Sport Science and Sport Friedrich‐Alexander‐Universität Erlangen‐Nürnberg Erlangen Germany

**Keywords:** children, high intensity, interval training, youth

## Abstract

This meta‐analysis examined whether HIIT outperforms low‐intensity endurance training (LIET), generic training (GT), or sport‐specific drills (SSD) in improving aerobic and anaerobic endurance in youth athletes. Systematic searches of PubMed, MEDLINE, SPORTDiscus, and Web of Science identified studies comparing HIIT with LIET, GT, or SSD in athletes aged ≤18 years. Outcomes included maximal oxygen uptake (VO_2_max), continuous endurance, intermittent endurance, and repeated sprint ability. Random‐effects meta‐analyses computed standardized mean differences, with risk of bias and sensitivity analyses conducted. Twenty‐eight studies (*N* = 707, 10.7% female) met inclusion criteria. HIIT elicited significantly greater VO_2_max improvements than GT (*g* = 0.97; *p* < 0.01) and also surpassed GT for continuous endurance performance (*g* = 0.91; *p* < 0.01). Compared to LIET, HIIT achieved similar VO_2_max and continuous endurance outcomes despite less total training time. Against SSD, HIIT improved continuous endurance performance more (*g* = 0.41; *p* = 0.02), while showing no difference in VO_2_max, intermittent endurance, or repeated sprint ability. HIIT is an effective, time‐efficient modality for enhancing aerobic capacity and continuous endurance performance in youth athletes. It outperforms GT and is at least as effective as LIET and SSD. Coaches should consider incorporating HIIT into youth training programs to optimize endurance adaptations within limited training time. Future research should investigate long‐term effects, consider female representation, and evaluate distinct HIIT protocols to further refine evidence‐based training guidelines.

## 1 INTRODUCTION

High‐intensity interval training (HIIT) has garnered substantial attention as a versatile and effective training modality for enhancing performance and health across diverse populations, including elite (Stöggl & Sperlich, 2015) athletes, recreational exercisers (Gillen & Gibala, 2014), and individuals with chronic diseases (Kessler et al., 2012). The appeal of HIIT lies in its ability to produce significant improvements in both aerobic and anaerobic performance within a relatively short period, positioning it as an efficient alternative to traditional moderate‐intensity continuous training (MICT) (Laursen & Jenkins, 2002). While the benefits of HIIT are well documented in adult populations, particularly regarding cardiovascular fitness, metabolic health, and sport‐specific performance (Gibala et al., 2012; Weston et al., 2014), its application in younger populations, especially adolescent athletes, is comparatively underexplored (Eddolls et al., 2017; Engel et al., 2018).

Adolescence is a critical period for physical and athletic development, during which training adaptations can have long‐lasting effects on performance and health (Bergeron et al., 2015). Young athletes often engage in specialized training regimens designed to optimize their physiological and skill development. In this context, HIIT presents a potentially valuable training method, not only for enhancing endurance and anaerobic capacity but also for offering time‐efficient training strategies that allow for a greater emphasis on sport‐specific skills (Buchheit & Laursen, 2013a). However, the evidence supporting the efficacy of HIIT in youth athletes remains fragmented, particularly when compared to other training modalities, for example, low‐intensity endurance training (Costigan et al., 2016; Engel et al., 2018).

A notable attempt to consolidate the effects of HIIT on endurance and anaerobic performance in youth athletes was made by Engel and colleagues (Engel et al., 2018) but failed to differentiate between training modalities when comparing the effectiveness of HIIT. This lack of distinction complicates the interpretation of HIIT's efficacy, especially since some of the studies included in their analysis compared HIIT to other high‐intensity training modalities, such as sprint interval training or Small‐Sided Games (SSG) (Engel et al., 2018). These high‐intensity modalities share some physiological demands with HIIT, potentially confounding the conclusions drawn about the unique benefits of HIIT.

Another meta‐analysis (Kunz et al., 2019) directly compared HIIT with SSG, revealing that both enhance physical performance through distinct mechanisms, impacting performance factors differently. This highlights that generalizing HIIT's effectiveness without accounting for the comparison modality may lead to misleading conclusions, especially when both involve high‐intensity efforts. To address these challenges, the current study compares HIIT with three specific training modalities commonly used in youth athletic development: Generic training (GT, i.e., technical, tactical sport‐specific training), low‐intensity endurance training (LIET, i.e., long slow distance running), and sport‐specific drills (SSD, i.e., SSG in soccer/basketball, specific on‐court drills in tennis, or specific throwing drills in combat sports). Each offers distinct benefits and is widely employed in different sports contexts. By focusing on these comparisons, this study aims to clarify when and how HIIT can be most effectively applied in youth sports training.

Given the methodological gaps and narrow comparative focus of existing meta‐analyses on high‐intensity training in youth athletes, an updated, comprehensive evaluation is warranted. The main aim of this meta‐analysis, therefore, is to evaluate how HIIT compares to generic training, low‐intensity endurance training, and sport‐specific drills in enhancing aerobic and anaerobic performance in youth athletes. By addressing these comparisons, the study seeks to contribute to the ongoing dialogue on optimizing youth athletic training and provide evidence‐based recommendations for practitioners aiming to improve performance through targeted training strategies (Logan et al., 2014; Seiler & Tønnessen, 2009).

## 2 METHODS

### 2.1 Systematic literature search

**FIGURE 1:**
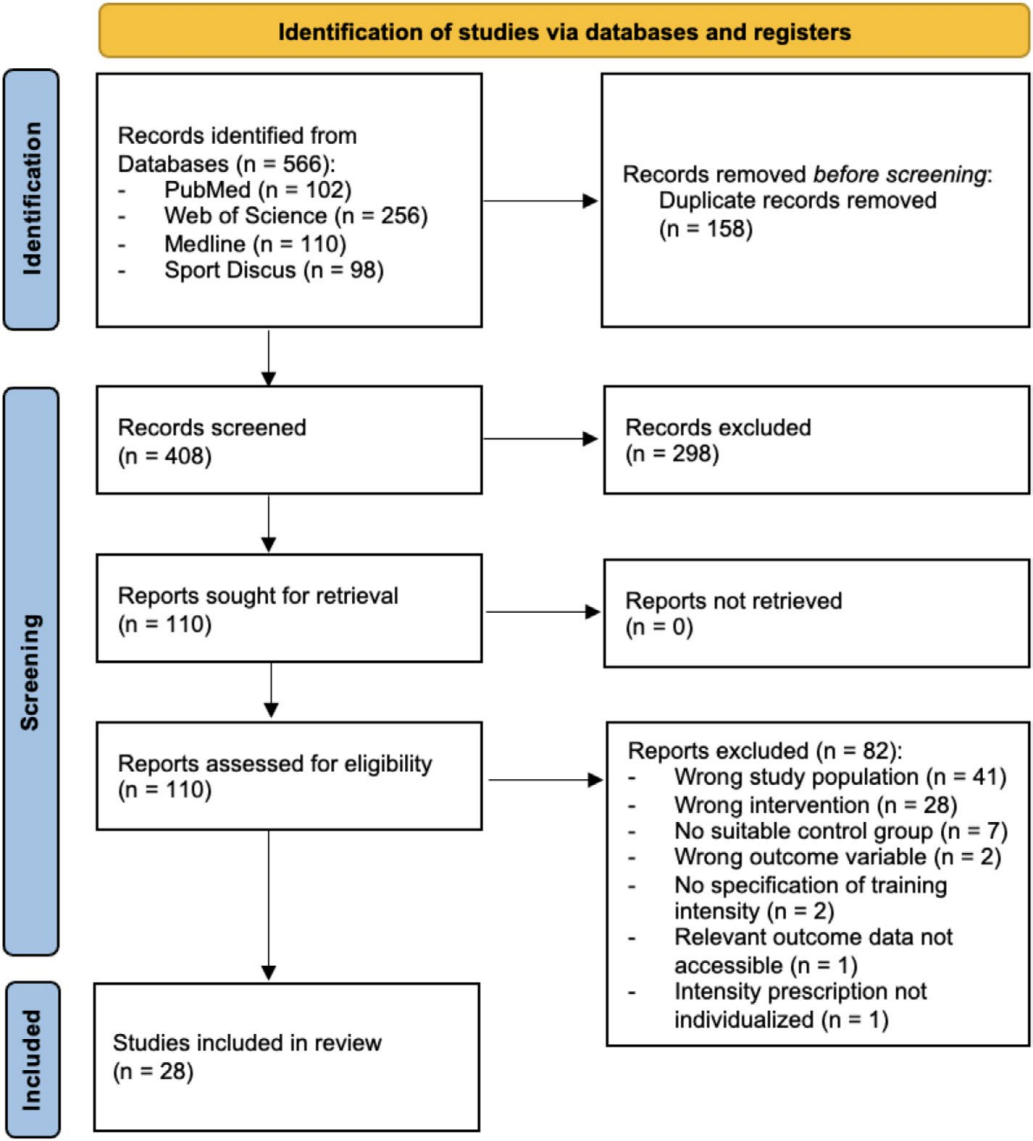
Prisma search flow chart.

A systematic literature search was conducted in accordance with the Preferred Reporting Items for Systematic Reviews and Meta‐Analyses (PRISMA) guidelines and was preregistered on the Open Science Framework (registration DOI: https://doi.org/10.17605/OSF.IO/PGNMH). We systematically searched the electronic databases PubMed, MEDLINE, SPORTDiscus, and Web of Science from their inception until June 10, 2024, for original, peer‐reviewed research articles in English that investigated the effects of HIIT on various endurance performance parameters (e.g., maximum oxygen uptake, time trial performance, repeated sprint ability) in youth athletes ≤18 years of age. Boolean search phrases were employed to identify studies relevant to the training intervention, age group, and athletic status. Keywords within each category were combined using the ‘OR’ operator, while the three categories were integrated using the “AND” operator (Table 1). Additionally, the reference lists of retrieved articles were screened to identify further relevant studies. For articles with missing essential information, the authors were contacted directly to obtain the required data. The entire literature search process—including record saving, duplicate removal, and the screening of titles, abstracts, and full texts—was conducted independently by two authors (MM and ML). Any disagreements were resolved through discussion until a consensus was achieved. The search strategy and study selection process are summarized in Figure 1.

**TABLE 1:** The terms, keywords, and phrases utilized in the present search of the scientific literature (Example for the search in PubMed).

Search term	Keywords
1. High‐intensity interval training	(“High‐Intensity Interval Training[Mesh] OR high intensity training” OR “intensive interval training” OR “high intensity circuit training” OR “aerobic interval training” OR “Circuit‐Based Exercise[Mesh] OR repeated sprint training” OR “intensive exercise”)
2. Young (≤18 years)	(Adolescent[Mesh] OR Child[Mesh])
3. Athletes	(Athletes[Mesh] OR Sports[Mesh])
Search phrase	1 AND 2 AND 3

### 2.2 Selection of relevant investigations

The original research articles selected for analysis were all published in international, peer‐reviewed medical or sports‐specific journals and all assessed the effects of some form of high‐intensity interval training performed by youth athletes (≤18 years) on maximum oxygen uptake, endurance running performance, intermittent running performance, and/or repeated sprint ability (Table 2). To avoid dependent effect estimates from the same sample, only the highest‐ranked outcome per category– based on test objectivity and physiological specificity– was included (see Table 2).

**TABLE 2:** Inclusion hierarchy of outcome measures^a^.

Outcome measures	Measurements
Maximum oxygen uptake	Maximum oxygen uptake (mL·kg^−1^·min^−1^ or mL·min^−1^) from respiratory gas analysis during an incremental step/ramp test
	Estimated maximum oxygen uptake (mL·kg^−1^·min^−1^) from intermittent running tests like YoYo‐IR1, 30–15 IFT etc
Endurance performance	Time trial performance, time to exhaustion at a fixed intensity or incremental step/ramp test
	Maximum aerobic speed from a shuttle run test
	Individual anaerobic threshold from an incremental step/ramp test
Intermittent endurance performance	Covered distance / total duration during an intermittent running test
	Maximum velocity during an intermittent running test
Repeated sprint ability	Mean time of a repeated sprint ability test

^a^ If studies provided various measurements for an outcome measure, only the one that is ranked highest in the hierarchy was included in the analysis.

### 2.3 Inclusion and exclusion criteria

Studies were considered for analyses when they included one or more of the following comparisons: (Stöggl & Sperlich, 2015) HIIT versus GT, where the GT was considered the normal training that was either partially replaced or substituted by HIIT in the intervention group, or as additional normal/low‐intensity sport‐specific technical/tactical training that was added to yield the same amount of training time as in the intervention group. (Gillen & Gibala, 2014) HIIT versus LIET, where LIET was considered low‐ to moderate‐intensity general endurance training using continuous, fartlek, or long‐interval exercise bouts. (Kessler et al., 2012) HIIT versus SSD, where SSD was considered sport‐specific high‐intensity interval routines, like Small‐Sided Games in team sports, on‐court drills in tennis, or throwing drills in combat sports.

Only randomized controlled trials with between‐group comparisons were eligible for inclusion. Studies were considered eligible if they met the following criteria: (Stöggl & Sperlich, 2015) prescribed HIIT defined as protocols involving ≥90% of maximal oxygen uptake (VO_2_max) (Buchheit & Laursen, 2013a), 90%–95% of peak heart rate (Sperlich et al., 2011), or supra‐maximal interval sprinting (Laursen & Jenkins, 2002). This included various HIIT modalities such as (a) long intervals (≥60 s), (b) short intervals (≤30 s), (c) repeated‐sprint training (RST) with efforts lasting 3–7 s and recovery periods generally less than 60 s, and (d) sprint interval training (SIT) consisting of 30‐s all‐out efforts interspersed with 2–4 min of passive recovery. (Gillen & Gibala, 2014) Involved male and female children or adolescents (≤18 years old) participating in any sport, assessing (a) maximum oxygen uptake, (b) endurance performance (i.e., time trial performance, maximum aerobic speed), (c) intermittent endurance performance (i.e., covered distance during Yo‐Yo IR1, 30–15 IFT) and/or (d) repeated sprint ability (cf. Table 2). (Kessler et al., 2012) Implemented an intervention duration of ≥4 weeks or a HIIT micro‐cycle of approximately 5–14 days, as defined by previous studies (Meckel et al., 2014; Wahl et al., 2013). (Laursen & Jenkins, 2002) Included a control group performing (a) low‐intensity endurance training, (b) generic training, or (c) sport‐specific games and (Gibala et al., 2012) participants were randomly assigned to the intervention or control group.

Training of the different control conditions was further defined as follows: (1) GT was considered the normal training that was either partially replaced or substituted by HIIT in the intervention group, or as additional normal/low‐intensity sport‐specific technical/tactical training that was added to yield the same amount of training time as in the intervention group. (2) LIET was considered low‐ to moderate‐intensity general endurance training using continuous, fartlek, or long‐interval exercise bouts. (3) SSD was considered sport‐specific high‐intensity interval routines, like SSG in team sports, on‐court drills in tennis, or throwing drills in combat sports.

Studies were excluded if they (1) involved youth not defined as athletes (e.g., sedentary individuals, pupils) or patients with conditions such as obesity, diabetes mellitus, or asthma; (2) focused solely on strength training; (3) did not provide detailed information on the HIIT intervention, including prescribed intervals and individualized intensities; (4) conducted interventions after detraining phases; (5) included a control group that performed alternative training without providing information on the protocol; (6) were conference abstracts, dissertations, theses, or articles published in non‐peer‐reviewed journals; or (7) implemented additional nutritional interventions or used ergogenic aids.

### 2.4 Data extraction

Two reviewers (ML and MM) independently evaluated the full‐text articles and extracted the data using a standardized, predefined form. In cases where consensus was not reached, a third reviewer (BS) was consulted to resolve disagreements. Collected data included authorship, year of publication, participant characteristics (age, sex, sample size, sport), details of the training interventions (frequency, duration, intensity, modality), and outcome measures before and after the intervention (see Table 2). When group means and standard deviations were not provided in the text or tables, we contacted the corresponding authors to request the baseline and post‐intervention data. If data were presented only in graphical form, we used the WebPlotDigitizer software (https://automeris.io/WebPlotDigitizer/) to extract numerical values by measuring the pixel lengths of the axes and data points to estimate means and standard deviations (Drevon et al., 2017).

### 2.5 Assessment of methodological quality

To evaluate the methodological quality of the included studies, two authors (MM and ML) independently assessed each study using the revised risk as recommended in the Cochrane Handbook for Systematic Reviews of Interventions (Higgins et al., 2024; Sterne et al., 2019). The risk of bias was assessed in six key domains of bias: bias arising from the randomization process, bias due to deviations from intended interventions, bias due to missing outcome data, bias in the measurement of the outcome, bias in the selection of the reported result and overall bias. Each domain was rated as “low risk,” “some concerns,” or “high risk,” based on the criteria outlined in the tool.

Bias due to deviations from intended interventions, specifically related to the blinding of participants and personnel, was a significant consideration, particularly given the inherent challenges of blinding in exercise interventions. In most studies, blinding of participants was not feasible due to the nature of the interventions. However, the risk of performance bias was rated with consideration of the objective nature of the outcome measures, which were typically assessed using standardized protocols and equipment.

Any disagreements between the reviewers were resolved through discussion. If consensus could not be reached, a third reviewer (BS) was consulted. All ratings were subsequently subjected to a final review for consistency, following the detailed guidance provided in the RoB 2 tool's algorithm (Sterne et al., 2019).

### 2.6 Data synthesis and analysis

The study compared the effect of HIIT interventions versus LIET, GT, or SSD on different endurance performance parameters. Where studies presented multiple outcome measures for the same outcome (i.e., time trial performance and maximum aerobic speed from a shuttle run test for endurance performance), only the one was included in the analysis that was highest according to the hierarchy shown in Table 2. As recommended by Cochrane (Higgins et al., 2024), this procedure avoids including statistically dependent intervention effects in the analysis, as they originate from the same participants.

Utilizing the means and standard deviations for the values of the intervention and control groups, the standardized mean difference (SMD) with 95% confidence intervals (CI) and 95% prediction intervals (PI) was calculated. The SMD was calculated as the mean pre‐post change in the treatment group minus the mean pre‐post change in the control group, divided by the pooled pre‐test standard deviation (see Formula 2 and 3) and adjusted for small sample sizes (Morris, 2007) (see Formula 3).

(1) $$d_{\mathrm{ppc}2}=c_{p}\;\frac{\left(M_{\text{post},T}-M_{\mathrm{pre},T}\right)-\left(M_{\text{post},C}-M_{\mathrm{pre},C}\right)}{SD_{\text{pooled},\mathrm{pre}}}$$

(2) $$SD_{\text{pooled},\mathrm{pre}}=\sqrt{\frac{\left(n_{T}-1\right)SD_{T,\mathrm{pre}}^{2}+\left(n_{C}-1\right)SD_{T,\mathrm{pre}}^{2}}{n_{T}+n_{C}-2}}$$

(3) $$c_{p}=1-\frac{3}{4\left(n_{T}+n_{C}-2\right)-1}$$

Heterogeneity (i.e., *τ* ^2^) was evaluated utilizing the restricted maximal‐likelihood estimator (Viechtbauer, 2005) and these analyses complemented with the *Q*‐test for heterogeneity (Cochran, 1954) and the *I* ^2^ statistical analysis (Higgins & Thompson, 2002) to determine the proportion of the total variability due to between‐study heterogeneity.

Sensitivity analyses were performed to assess the robustness of our findings and to identify potential outliers and influential studies. Outliers were detected using externally studentized residuals with a Bonferroni‐adjusted significance level to account for multiple comparisons (Viechtbauer & Cheung, 2010). Specifically, for *k* studies, a study was considered an outlier if its studentized residual exceeded the critical value $z_{\text{crit}}=\Phi^{-1}\left(1-\frac{\alpha }{2k}\right)$ where $\Phi^{-1}$ is the inverse cumulative distribution function of the standard normal distribution and α=0.05.

Influential studies were identified based on Cook's distances. To establish a robust cutoff, we calculated the median and interquartile range (IQR) of the Cook's distances across all studies. A study was deemed influential if its Cook's distance exceeded the threshold defined as the median plus six times the IQR ($\text{Cuthoff}={\text{Median}}_{\mathrm{CD}}+6\times {\mathrm{IQR}}_{\mathrm{CD}}$) (Viechtbauer & Cheung, 2010). This method reduces the influence of extreme values and skewed distributions, providing a more reliable identification of studies that could disproportionately affect the meta‐analysis results.

To assess the potential for publication bias, we created funnel plots for each meta‐analysis (Sterne et al., 2011).

By applying these criteria, we aimed to minimize the impact of potential outliers and influential studies, ensuring that our conclusions regarding the effects of high‐intensity interval training on endurance performance outcomes were robust and not unduly influenced by any single study.

Analyses were carried out using RStudio (version 2024.09.0) (R Core Team R, 2020) and the metaphor software (version 4.6) (Viechtbauer, 2010). Statistical significance was determined using a significance level of *α* = 0.05.

## 3 RESULTS

### 3.1 Study characteristics

The comprehensive database search identified a total of 566 articles. After removing duplicates and excluding review articles and irrelevant titles, 298 articles were excluded, leaving 110 articles for further screening of abstracts and full texts. Ultimately, 28 studies (Arslan et al., 2020, 2022; Aschendorf et al., 2019; Breil et al., 2010; Buchheit et al., 2009; Delextrat & Martinez, 2014; Faude et al., 2013, 2014; Fernandez‐Fernandez et al., 2017; Hammami et al., 2021; Harrison et al., 2015; Helgerud et al., 2001; Hill‐Haas et al., 2009; Impellizzeri et al., 2006, 2008; Jastrzebski et al., 2014; Ketelhut et al., 2020; Kilit & Arslan, 2019; Los Arcos et al., 2015; Ouergui et al., 2020; Sandbakk et al., 2011, 2013; Seo et al., 2019; Sommer Jeppesen et al., 2022; Sperlich et al., 2010, 2011; Thom et al., 2020; Tonnessen et al., 2011) met the inclusion criteria and provided the necessary data for the current meta‐analysis. Of these, 17 studies (Buchheit et al., 2009; Delextrat & Martinez, 2014; Faude et al., 2013, 2014; Fernandez‐Fernandez et al., 2017; Harrison et al., 2015; Helgerud et al., 2001; Hill‐Haas et al., 2009; Impellizzeri et al., 2006, 2008; Los Arcos et al., 2015; Sandbakk et al., 2011, 2013; Sperlich et al., 2010, 2011; Tonnessen et al., 2011) were previously included in a meta‐analysis examining the effects of HIIT on performance parameters in youth athletes (Engel et al., 2018). A comprehensive overview of the participants, sports, prescribed training interventions, and outcome measures for each included study is highlighted in Tables 3, 4 and 5 for HIIT versus LIET, GT, and SSD, respectively.

**TABLE 3:** Overview of included studies comparing high‐intensity interval training to low‐intensity endurance training.

Author (year)	Group	Sex	*N*	Age [y]	Sport	Dura‐tion	Modality	Sess‐ Ions [*n*]	Frequ‐ency	Protocol	Prescribed intensity	Total time/session	Training load	Outcome measure
Faude et al. (2013)	INT	m	20	15.9 ± 0.8	Soccer	5.5 wks	Running	12–15	2–3/wk	2 × (12–15 × 15″:15″ or 20″:20″ or 30″:30″); 10′ rest	125% or 130% or 140% of vIAT	22–40′ (∅ 33′)	73.4 ± 3.6% HR_peak_	vIAT
	CON		20							30–60′ continous or fartlek runs	80%–95% vIAT	30–60′ (∅ 47′)	77.7 ± 4.4% HR_peak_	
Ketelhut et al. (2020)	INT	m	10	15.4 ± 1.30	Rowing	8 wks	Rowing	16	2/wk	2 × (4 × 2′; 1′ rest); 7′ rest	95% HR_max_	29′		TTE, VO_2max_
	CON		7	15.3 ± 1.21						70–90′	70% HR_max_	70–90′		
Sandbakk et al. (2011)	INT	m & f	7 (f = 2)	17.4 ± 0.5	XC‐Ski	8 wks	XC‐Ski	−/−	−/−	Multipe 5–10′ or 1–5′ Intervals; rest: n.a.	85%–92% or > 92% HR_max_	−/−	≤ 85% HR_max_: 6:57 h > 85% HR_max_: 2:08 h	TT, VO_2max_
	CON		8 (f = 3)							1 × 1.5–3 h or 1–2 h	60%–74% or 75–85% HR_max_	1.5–3 h	≤ 85% HR_max_: 8:22 h > 85% HR_max_: 1:21 h	
Sandbakk et al. (2013)	INT‐1	m & f^a^	7	17.5 ± 0.4	XC‐Ski	8 wks	XC‐Ski	16	2/wk	Multipe 2–4′ intervals, rest: n.a.	max. sustainable intensity (>85% HR_max_)	15–20′	≤ 85% HR_max_: 6:31 h > 87% HR_max_: 1:40 h	TT, VO_2max_
	INT‐2		7							Multiple 5–10′ intervals, rest: n.a.		40–45′	≤ 85% HR_max_: 6:57 h > 87% HR_max_: 2:17 h	
	CON		7							1′ 1.5–3 h	60%–74% HR_max_	1.5–3 h	≤ 85% HR_max_: 8:13 h > 87% HR_max_: 1:24 h	
Sperlich et al. (2010)	INT	m & f	26 (f = 13)	10.5 ± 1.4	Swim‐ming	5 wks	Swim‐ming	25	5/wk	Multiple intervals of 50–300 m	92% of personal best time	30′	sRPE_Borg_: 18.5 ± 1.5 BL_av_: 7.0 ± 1.5 mmol•L^−1^	TT, VO_2max_
	CON		26 (f = 13)							Multiple intervals of 100–800 m	85% of personal best time	60′	sRPE_Borg_: 13.7 ± 2.4 BLa_av_: 2.0 ± 0.8 mmol•L^−1^	
Sperlich et al. (2011)	INT	m	9	13.5 ± 0.4	Soccer	5 wks	Running	13	2–3/wk	4–12 × 30″‐4′ intervals, 30″‐3′ active rest	90%–95% HR_max_	25–31′ (∅28.8)	60%–80% HR_max_: 35.9% 80%–90% HR_max_: 35.8% 90%–100% HR_max_: 28.3%	TT, VO_2max_
	CON		8							2–5 × 10–30′ Fartlek, 1–5′ rest or continuous runs	50%–70% HR_max_	51–69′ (∅57.3)	60%–80% HR_max_: 71.9% 80%–90% HR_max_: 26.0% 90%–100% HR_max_: 2.1%	

Abbreviations: CON, control group; f, female; HR_max_, maximum heart rate; HR_peak_, peak heart rate; hrs, hours; INT, intervention group; m, male; TT, time trial; TTE, time to exhaustion; vIAT, velocity at individual anaerobic threshold; VO_2max_, maximum oxygen uptake; wk., week; wks, weeks.

^a^ The authors report no differences between sex and therefore do not provide information on the proportion of male and female athletes between groups.

**TABLE 4:** Overview of included studies comparing high‐intensity interval training to generic training.

Author (year)	Group	Sex	*N*	Age [y]	Sport	Dura‐tion	Modality	Sess‐ions [*n*]	Frequ‐ency	Protocol	Prescribed Intensity	Total time/session	Training load	Outcome measure
Aschendorf et al. (2018)	INT^a^	f	11	15.1 ± 1.1	Basketball	4 wks	Basketball	8	2/wk	Session A: 4 × 4′; 3′ rest Session B: 2 × (2 × 30″; 15″ rest); 3′ rest	90%–95% HR_max_	25′	−/−	Yo‐YoIR1
	CON		13				−/−	−/−	−/−	Continued with their normal team practice	−/−	−/−	−/−	
Breil et al. (2010)	INT^b^	m & f	13 (f:4)	17.4 ± 1.1	Alpin Ski	11 days shock cylce	Cycling (12×) Running (3×)	15	5‐10/wk	4 × 4′; 3′ rest	90%–95% HR_max_	25′	Endurance Tr.: 10.4 h Strength Tr.: 2.7 h Total Tr.: 13.9 h TRIMP: 6328 a.u.	VO_2max_, Tlim
	CON		8 (f:2)	16.6 ± 1.1			−/−	−/−	−/−	Normal endurance and strength training	−/−	−/−	Endurance Tr.: 9.1 h Strength Tr.: 8.6 h Total Tr.: 23.9 h TRIMP: 8119 a.u.	
Hammami et al. (2021)	INT^b^	m	17	16.6 ± 0.5	Handball	8 wks	Running	16	2/wk	4 × (6 × 5″; 10″ rest); 3–5′ rest	130% MAS	25–30′	−/−	eVO_2max_, RSA, MAS
	CON		15				−/−	−/−		Habitual fitness sessions; technical/tactical training	−/−	25–35′	−/−	
Helgerud et al. (2001)	INT^a^	m	9	18.1 ± 0.8	Soccer	8 wks	Running	16	2 /wk	4 × 4′; 3′ active rest	90%–95% HR_max_	25′	−/−	VO_2max_
	CON^a^		10				−/−			Additional technical training	−/−	−/−	−/−	
Impellizzeri et al. (2008)	INT^a^	m	11	17.8 ± 0.6	Soccer	4 wks	Running	11	2‐3/wk	4 × 4′, 3′ rest	90%–90% HR_max_	25′	TRIMP_sRPE_: 552 a.u.	VO_2max_
	CON^a^		10				Soccer			Additional technical and tactical training	−/−	25′	TRIMP_sRPE_: 253 a.u.	
Ouergui et al. (2020)	INT^a^	m & f	12 (f:3)	16 ± 1	Judo	4 wks	Running	8	2/wk	3–6 × (10 × 35 m; 10″ rest); 3′ rest	All‐out	13–30′	TRIMP_sRPE_: 607 a.u.	eVO_2ma_
	CON		12 (f:3)	16 ± 1			−/−	−/−	−/−	Same taekwondo training sessions as HIIT group without additional training	−/−	−/−	TRIMP_sRPE_: 617 a.u.	
Seo et al. (2019)	INT^a^	m	36	16.7 ± 0.84	Teakwondo	4 wks	Running	10	2‐3/wk	Gr1: 6–8 × 30″; 1′ rest Gr2: 6–8 × 30″; 2′ rest Gr3: 6–8 × 30″; 4′ rest	90%–100% HR_max_	9–12′ 15–20′ 27–36′	Gr1: HR = 157.5–187.1 bpm Gr2: HR = 132.6–187.9 bpm Gr3: HR = 127.6–187.1 bpm	VO_2max_, TTE
	CON		11				−/−	−/−		Regular teakwondo exercise	−/−	−/−	−/−	
Sommer Jeppesen et al. (2022)	INT^b^	m	17	18 ± 1.0	Ice Hockey	4 wks	Ice Skating	12	3/wk	Final 15–25′of on‐ice training substituted with: 6–10 × 20″, 2′ rest	>95% v_max_	15–25′	∅HR: 83%Hrmax Weekly training time: 04:34 h	YoYo‐IR1, VO_2max_
	CON		17				Ice Hockey			Regular on‐ice training	−/−	15–25′	∅HR: 78%Hrmax Weekly training time: 04:32 h	
Thom et al. (2019)	INT^a^	m	7	15 ± 0.6	Soccer	6 wks	Cycling	12	2/wk	6 × 10″; 80″ rest	All‐out	9′	−/−	VO_2peak_, TTE
	CON		6	15 ± 0.5			−/−	−/−	−/−	Maintained their normal training activities	−/−	−/−	−/−	
Tonnessen et al. (2011)	INT^a^	m	10	16.4 ± 60.9	Soccer	10 wks	Running	10	1	2–5 × (4–5 × 40 m; 90″ rest); 10′ rest	95%–100% v_max_	20–65‘	−/−	RSA
	CON		10				−/−	−/−	−/−	Continued the teams' original training plan	−/−	−/−	−/−	

Abbreviations: a.u., arbitrary units; bpm, beats per minute; CON, control group; eVO_2max_, estimated maximum oxygen uptake; f, female; HR, heart rate; HR_max_, maximum heart rate; Hrmax, maximum heart rate; hrs, hours; INT, intervention group; m, male; MAS, maximum aerobic speed; RSA, repeated sprint ability; sRPE, session Rating of Perceived Exertion; Tlim, time to exhaustion; Tr., training; TRIMP, training impulse; TTE, time to exhaustion; v_max_, maximum velocity; VO_2max_, maximum oxygen uptake; VO_2peak_, peak oxygen uptake; wk., week; wks, weeks; YoYo‐IR1, Yo‐Yo Intermittent Recovery Test Level 1.

^a^ Training was added to normal training.

^b^ Training replaced parts of the normal training; no subscripted letter = normal training was unaltered.

**TABLE 5:** Overview of included studies comparing high‐intensity interval training to sport‐specific drills.

Author (year)	Group	Sex	*N*	Age	Sport	Dura‐Tion	Modality	Sess‐ions [*n*]	Frequ‐ency	Protocol	Prescribed intensity	Total time/session	Training load	Outcome measure
Arslan et al. (2022)	INT	m	16	14.6 ± 0.5	Basketball	6 wks	Running	18	3/wk	2 × (6–9′ of 15″:15″ intervals); 2′ rest	90%–95% vIFT	14–20′	RPE: 8.5–9.1	vIFT, YYIRT‐1, eVO_2max_, RSA
	CON		16	14.4 ± 0.4			Basketball			2 × 5–8′; 2′ rest	−/−	12–18′	RPE: 7.4–8.0	
Arslan et al. (2020)	INT	m	10	14.1 ± 0.6	Soccer	5 wks	Running	10	2/wk	2 × (6–10′ of 15″:15″ intervals); 2′ rest	90%–95% vIFT	14–22′	RPE: 18.3	vIFT, TT, YYIRT‐1, eVO_2max_, RSA
	CON		10	14.4 ± 0.5			Soccer			2 × 5–9′; 2′ rest	−/−	12–20′	RPE: 16.6	
Buchheit et al. (2009)	INT	m & f	17 (f = 8)	m: 15.7 ± 0.9 f: 15.2 ± 0.9	Handball	10 wks (1 rest week)	Running	17	2/wk	m: 6:15–12:15′ of 15″:15″ intervals f: 5:30–11:15′ of 15″:15″ intervals	m: 92–100% vIFT f: 90%–95% vIFT	m: 6:15–12:15′ f: 5:30–11:15′	1st session: 87.6% HR_max_ 17th session: 86.6% HR_max_	vIFT, Tlim_100%vIFT_, RSA
	CON		15 (f = 8)				Handball			m: 2–3 × 2:45–4:15′; 30″ rest f: 2–3 × 2:30–3:20′; 30″ rest	−/−	m: 6–12′ f: 5:30–11′	1st session: 86.8% HR_max_ 17th session: 85.9% HR_max_	
Delextrat and Martinez (2014)	INT	m	9	16.0 ± 0.6	Basketball	6 wks	Running	12	2/wk	8:00–13:00′ of 15″:15″ intervals	95% vIFT	8–13′	90.5% of HR_peak_	VIFT, RSA
	CON		9	16.3 ± 0.8			Basketball			2–3 × 3:00–4:15′	−/−	7:30–12′	90.6% of HR_peak_	
Faude et al. (2014)	INT	m	19	16.5 ± 0.8	Soccer	4 wks	Running	8	2/wk	2 × (12–15 × 15″; 15″ rest); 10′ rest	40% > IAT	21:30–24:30′	Max. HR: 98.5% HR_max_ ∅HR: 73.0% HR_max_	IAT
	CON		19				Soccer			4 × 4’; 3′ rest	−/−	28′	Max. HR: 95.5% HR_max_ ∅ HR: 72.7% HR_max_	
Fernandez‐Fernandez et al. (2017)	INT^A^	m	8	14.8 ± 0.1	Tennis	8 wks	Running, Tennis	16	2/wk	Running: 2 × (15–22 × 15″; 15″ rest); 3′ rest Tennis: 1 × (2 × 8–11′); 3′ rest	90%–95% vIFT −/−	19–25′	RPE: 7.2 TRIMP: 148.0 a.u.	VO_2max_, vIFT
	CON^A^		9				Tennis			1 × (2 × 8–11′); 3′ rest	−/−	19–25′	RPE: 6.4 TRIMP: 127.4 a.u.	
Harrison et al. (2015)	INT	m	11	13.9 ± 0.3	Field hockey & Rugby	6 wks	Running	6	1/wk	2 × (16–22 × 15″; 15″ rest); 3′ rest	90%–95% vIFT	19–26′	TRIMP: 406.5 a.u./min	vIFT, VO_2max_
	CON		10				Field hockey & Rugby			2 × 8′; 3′ rest	−/−	19–26’	TRIMP: 321.1 a.u./min	
Hill‐Haas et al. (2009)	INT	m	9	14.6 ± 0.9	Soccer	7 wks	Running	14	2/wk	Various interval protocols	>90% HR_max_ ‐ Intensity_max_	43–75′/wk	< 80% HR_max_: 235‘ 80%–89% HR_max_: 117‘ >90% Hrmax: 65‘	TTE, YYIRT‐1, RSA, VO_2max_
	CON		10				Soccer			2–6 × 6–13′; 1–3′ rest	−/−	43–75′/wk	< 80% HR_max_: 195‘ 80%–89% HR_max_: 118‘ >90% HR_max_: 78‘	
Impellizzeri et al. (2006)	INT	m	29	17.2 ± 0.8	Soccer	12 wks	Running	24	2/wk	4 × 4′, 3′ active rest	90%–95% HR_max_	25′	∅ HR: 90.7% HR_max_	VO_2max_
	CON						Soccer			4 × 4′, 3′ rest	−/−		∅ HR: 91.3% HR_max_	
Jastrzebski et al. (2014)	INT	m	11	15.8 ± 0.55	Soccer	8 wks	Running	16	2/wk	7 × (6 × 15″, 15″ rest), 90″ rest	85%–90% HR_max_	30′	∅ HR: 88.8% HR_max_	VO_2max_
	CON		11	15.8 ± 0.63			Soccer			7 × 3′, 90″ rest	−/−	30′	∅ HR: 90.0% HR_max_	
Kilit & Arslan (2019)	INT	m	14	13.8 ± 0.4	Tennis	6 wks	Running	16	2‐3/wk	6–12 × 30″, 30″ rest, followed by 1–2 × 1–2′, 1–2′ rest	>85% HR_max_	8–16′	80%–100% HR_max_: 60.0%	TT, VO_2max_
	CON		15				Tennis			6–12 × 30″, 30″ rest, followed by 1–2 × 1–2′, 1–2′ rest	−/−	8–16′	80%–100% HR_max_: 64.3%	
Los Arcos et al. (2015)	INT	m	8	15.8 ± 0.5	Soccer	6 wks	Running	11	1‐2/wk	3 × 4′, 3′ active rest	90%–95% HR_max_	18′	> 90% HR_max_: 7.2% 80%–90% HR_max_: 22.6% < 80% HR_max_: 70.3%	MAS
	CON		7	15.1 ± 0.7			Soccer			3 × 4′, 3′rest	−/−	18′	> 90% HR_max_: 12.7% 80%–90% HR_max_: 19.4% < 80% HR_max_: 68.0%	
Ouergui et al. (2020)	INT	m & f	12 (f:3)	16 ± 1	Teakwondo	4 wks	Running	8	2/wk	3–6 × (10 × 35 m; 10″ rest); 3′ rest	All‐out	−/−	TRIMP_sRPE_: 607 a.u.	eVO_2max_
	CON		12 (f:3)	16 ± 1			Teakwondo			10 × 6″ AMRAP of taekwondo technique, 10″ rest	−/−	−/−	TRIMP_sRPE_: 624 a.u.	

Abbreviations: %vIFT, % of the velocity reached at the end of the 30–15 Intermittent Fitness Test; a.u., arbitrary units; CON, control group; eVO_2max_, estimated maximum oxygen uptake; f, female; HR, heat rate; HR_max_, maximum heart rate; HR_peak_, peak heart rate; IAT, individual anaerobic threshold; INT, intervention group; Intensity_max_, maximum intensity; m, male; MAS, maximum aerobic speed; Max. HR, Maximum heart rate; min, minutes; RPE, Rating of Perceived Exertion; RSA, repeated sprint ability; Tlim_100_, times‐to‐exhaustion running at 100% of VIFT; TRIMP, training impulse; TRIMP_sRPE_, TRIMP session Rating of Perceived Exertion; TT, time trial; TTE, time to exhaustion; vIFT, maximum speed reached in the last stage of the 30–15 Intermittent Fitness Test; VO_2max_, maximum oxygen uptake; wk., week; wks, weeks; YYIRT‐1, Yo‐Yo Intermittent Recovery Test Level 1.

The meta‐analysis encompassed a total of 707 youth athletes, of whom 76 (10.7%) were female. Specifically, data were available for comparisons of HIIT versus GT in 10 studies (Aschendorf et al., 2019; Breil et al., 2010; Hammami et al., 2021; Helgerud et al., 2001; Impellizzeri et al., 2008; Ouergui et al., 2020; Seo et al., 2019; Sommer Jeppesen et al., 2022; Thom et al., 2020; Tonnessen et al., 2011), SSD in 13 studies (Arslan et al., 2020, 2022; Buchheit et al., 2009; Delextrat & Martinez, 2014; Faude et al., 2014; Fernandez‐Fernandez et al., 2017; Harrison et al., 2015; Hill‐Haas et al., 2009; Impellizzeri et al., 2006; Jastrzebski et al., 2014; Kilit & Arslan, 2019; Los Arcos et al., 2015; Ouergui et al., 2020), and LIET in 6 studies (Faude et al., 2013; Ketelhut et al., 2020; Sandbakk et al., 2011; Sperlich et al., 2010, 2011). These comparisons included 255 athletes (36 females, 14.1%) for HIIT versus GT, 316 athletes (22 females, 7.0%) for HIIT versus SSD, and 136 athletes (18 females, 13.2%) for HIIT versus LIET.

The mean age of the athletes included in the meta‐analysis was 15.7 ± 1.4 years, with an age range from 10.5 to 18.1 years and a median age of 15.8 years. The included studies spanned various sports disciplines: soccer (*n* = 12) (Arslan et al., 2020; Faude et al., 2013, 2014; Helgerud et al., 2001; Hill‐Haas et al., 2009; Impellizzeri et al., 2006, 2008; Jastrzebski et al., 2014; Los Arcos et al., 2015; Sperlich et al., 2011; Thom et al., 2020; Tonnessen et al., 2011), basketball (*n* = 3) (Arslan et al., 2022; Aschendorf et al., 2019; Delextrat & Martinez, 2014), taekwondo (*n* = 2) (Ouergui et al., 2020; Seo et al., 2019), handball (*n* = 2) (Buchheit et al., 2009; Hammami et al., 2021), tennis (*n* = 2) (Fernandez‐Fernandez et al., 2017; Kilit & Arslan, 2019), cross‐country skiing (*n* = 2) (Sandbakk et al., 2011, 2013), alpine skiing (*n* = 1) (Breil et al., 2010), ice hockey (*n* = 1) (Sommer Jeppesen et al., 2022), rowing (*n* = 1) (Ketelhut et al., 2020), swimming (*n* = 1) (Sperlich et al., 2010) and both field hockey and rugby (*n* = 1) (Harrison et al., 2015).

### 3.2 Risk of bias assessment

**FIGURE 2:**
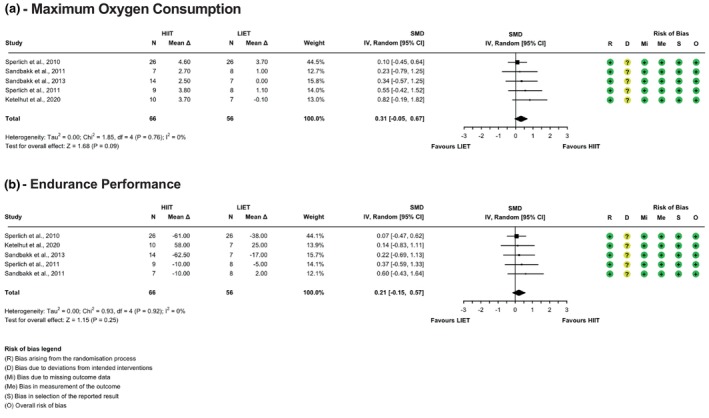
Forest plots with risk of bias assessment for HIIT versus low‐intensity endurance exercise on maximum oxygen consumption (a), continuous endurance performance (b).

**FIGURE 3:**
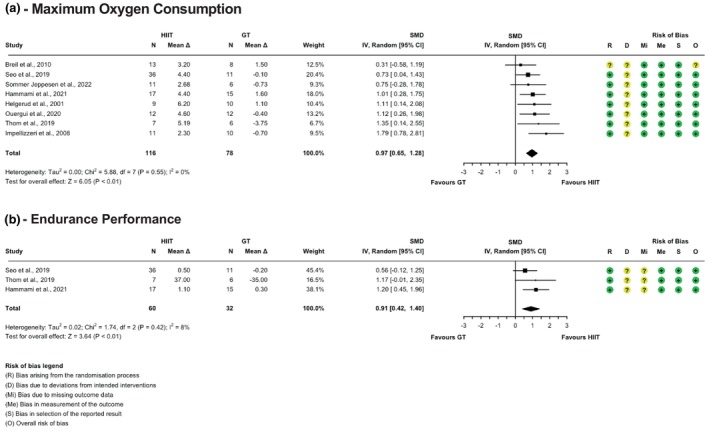
Forest plots with risk of bias assessment for HIIT versus generic sports training on maximum oxygen consumption (a) and continuous endurance performance (b).

**FIGURE 4:**
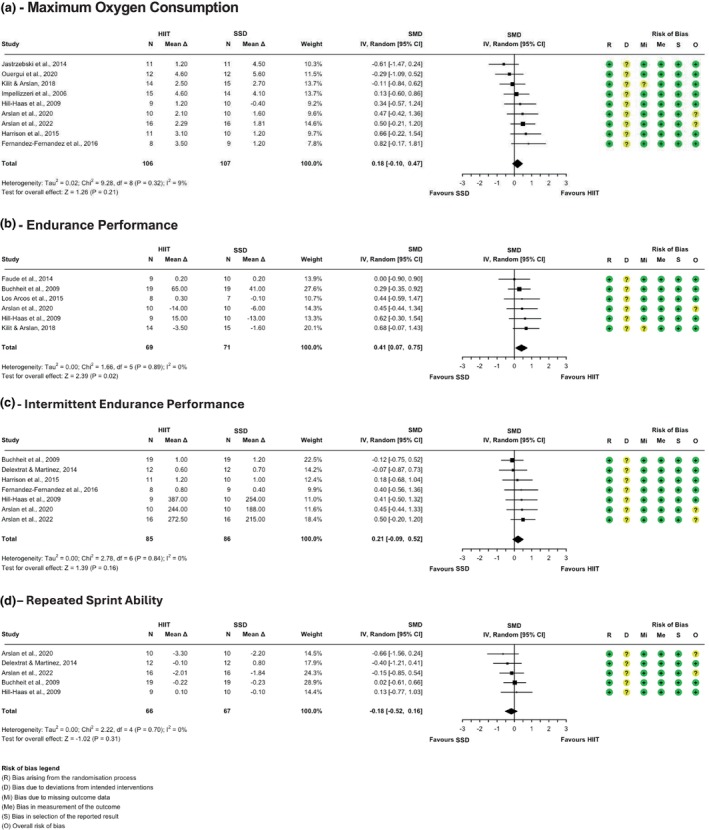
Forest plots with risk of bias assessment for HIIT versus sport‐specific drills on maximum oxygen consumption (a), continuous endurance performance (b), intermittent endurance performance (c), and repeated sprint ability (d).

Random sequence generation was robust in approximately 93% of studies, though allocation concealment remained unclear in about 18% (cf., Figures 2, 3, 4). Blinding presented a consistent challenge across the studies, a common issue in exercise interventions where the observable nature of the intervention makes participant and trainer blinding impractical. This led to some concerns about deviations from intended interventions, although these did not appear to influence outcomes significantly. Bias due to missing outcome data and in the measurement of the outcome data was low, as all included studies reported complete outcome data and used objective, standardized measures. Selective reporting bias was also low, as all studies adhered to pre‐specified analysis plans. Overall, while the risk of bias was low in most domains, the challenges of blinding in exercise studies and potential performance bias require careful consideration when interpreting the results.

### 3.3 Effects of HIIT versus low‐intensity endurance training

Forest plots with risk of bias assessment for HIIT versus LIET are shown in Figure 2. In the comparison between HIIT and LIET on maximum oxygen consumption [VO_2_max], five studies were included. The random‐effects meta‐analysis revealed no significant difference between HIIT and LIET in improving VO_2_max (Hedges' *g* = 0.31; 95% CI: −0.05 to 0.67; *p* = 0.09). No heterogeneity was observed (*τ* ^2^ = 0.00; *I* ^2^ = 0.00%; Q(3) = 1.85; *p* = 0.76), and no outliers or influential studies were identified.

For endurance performance, six studies were included. The random‐effects meta‐analysis revealed no difference between HIIT and LIET in improving endurance performance (Hedges' *g* = 0.06; 95% CI: −0.25 to 0.37; *p* = 0.71). One influential study was identified based on cook's distance analysis (Faude et al., 2013). Excluding this outlier in a sensitivity analysis changed the effect size towards favoring HIIT more, but again, this effect is not statistically significant (Hedges' *g* = 0.21; 95% CI: −0.15 to 0.57; *p* = 0.25). No heterogeneity was observed (*τ* ^2^ = 0.00; *I* ^2^ = 0.00%; Q(3) = 0.93; *p* = 0.91).

### 3.4 Effects of HIIT versus generic training

Forest plots with risk of bias assessment for HIIT versus GT are shown in Figure 3. When comparing HIIT with GT on VO₂max, eight studies were analyzed. The meta‐analysis indicated a significant positive effect of HIIT over GT (Hedges' *g* = 0.97; 95% CI: 0.65 to 1.28; *p* < 0.01). No heterogeneity was observed (*τ* ^2^ = 0.00; *I* ^2^ = 0.00%; Q(7) = 5.95; *p* = 0.55), and no outliers or influential studies were identified.

For endurance performance, four studies compared HIIT with GT. The meta‐analysis showed that HIIT did not significantly differ from GT in improving endurance performance (Hedges' *g* = 0.59; 95% CI: −0.19 to 1.37; *p* = 0.14). One outlier was identified based on studentized residuals. Excluding this outlier in a sensitivity analysis increased the effect size (Hedges' *g* = 0.91; 95% CI: 0.42 to 1.40; *p* < 0.01) and low heterogeneity (*τ* ^2^ = 0.02; *I* ^2^ = 8%; Q(2) = 1.74; *p* = 0.42), indicating a significant effect of HIIT over GT.

### 3.5 Effects of HIIT versus sport‐specific drills

Forest plots with risk of bias assessment for HIIT versus SSD are shown in Figure 3. In the analysis between HIIT and SSD VO₂max, eight studies were included, and the meta‐analysis found no significant difference between HIIT and SSD (Hedges' *g* = 0.13; 95% CI: −0.16 to 0.42; *p* = 0.38). No outliers or influential studies were identified, and heterogeneity was low (*τ* ^2^ = 0.01; *I* ^2^ = 3%; Q(7) = 7.56; *p* = 0.37).

For endurance performance, six studies compared HIIT and SSD, revealing a significant difference (Hedges' g = 0.41; 95% CI: 0.07 to 0.75; *p* = 0.02). No outliers or influential studies were identified, and no heterogeneity was observed (*τ* ^2^ = 0.00; *I* ^2^ = 0.00%; Q(5) = 1.66; *p* = 0.02).

For intermittent performance, six studies were analyzed. The meta‐analysis showed no difference between the effectiveness of SSD and HIIT (Hedges' *g* = 0.19; 95% CI: −0.12 to 0.51; *p* = 0.23). No outliers or influential studies were identified, and no heterogeneity was observed (*τ* ^2^ = 0.00; *I* ^2^ = 0.00%; Q(5) = 2.63; *p* = 0.76).

In the comparison of HIIT and SSD on repeated sprint ability (RSA), five studies were included. The meta‐analysis indicated no significant difference between the two training modalities (Hedges' *g* = −0.18; 95% CI: −0.52 to 0.16; *p* = 0.31). No outliers or influential studies were identified, as well as no heterogeneity (*τ* ^2^ = 0.00; *I* ^2^ = 0.00%; Q(4) = 2.22; *p* = 0.70).

Funnel plots were visually inspected for asymmetry to assess potential publication bias (Supporting Information—S1). The plots appeared symmetrical for most analyses, suggesting a low risk of publication bias. Due to the relatively small number of studies in each meta‐analysis, statistical tests for funnel plot asymmetry were not performed, as they may lack sufficient power to detect bias in small samples.

## 4 DISCUSSION

The current meta‐analysis aimed to analyze the effects of HIIT compared to GT, LIET, and SSD in improving aerobic and anaerobic performance in youth athletes.

The results of this meta‐analysis indicate that HIIT is an effective training modality for enhancing VO_2_max and endurance performance in youth athletes. Specifically, HIIT significantly outperforms GT in improving VO_2_max and is equally effective compared to LIET. When compared to SSD, HIIT demonstrates superior improvements in endurance performance, although both HIIT and SSD are equally effective in enhancing VO_2_max.

While our findings align with earlier meta‐analyses in terms of general HIIT effectiveness, the present study advances the field by differentiating comparator types (GT, LIET, SSD) and including 11 additional studies published after the latest synthesis. This allows for more specific guidance on how HIIT compares to common training approaches in youth sport practice.

### 4.1 HIIT versus low‐intensity endurance training

When comparing HIIT with LIET interventions in youth athletes, no statistically significant differences in VO_2_max (Hedges' *g* = 0.31; 95% CI: −0.05 to 0.67; *p* = 0.09) or endurance performance (Hedges' *g* = 0.06; 95% CI: −0.25 to 0.37; *p* = 0.71) were found. Notably, these similar improvements were achieved despite the HIIT protocols requiring approximately half the total training time of the LIET protocols, highlighting the potential of HIIT as a time‐efficient alternative (see Table 3). The efficiency of HIIT likely reflects its capacity to simultaneously provoke central (e.g., augmented cardiac output and stroke volume) and peripheral (e.g., enhanced muscular oxidative capacity) adaptations (Buchheit & Laursen, 2013a). These physiological adaptations are driven by repeated high‐intensity efforts that increase oxidative enzyme activity and improve skeletal muscle oxygen utilization (Nicolo & Girardi, 2016).

For youth athletes, who frequently face competing academic and social demands, the reduced time commitment associated with HIIT is a considerable practical advantage. Addressing the common barrier of “lack of time” (Gillen & Gibala, 2014), HIIT may facilitate regular training adherence without compromising gains in aerobic capacity. Furthermore, younger athletes often prefer intermittent, variable‐intensity activities, which align with the inherent structure of HIIT (Barkley et al., 2009; Findlay et al., 2010; Leahy et al., 2020). This greater alignment with natural activity patterns may enhance enjoyment and motivation, fostering higher adherence rates and potentially superior long‐term outcomes (Burford et al., 2022; Eather et al., 2016). Although LIET remains a valuable and well‐established approach for enhancing endurance, these findings suggest that HIIT offers a viable, time‐efficient alternative that can support comparable performance adaptations.

It is important to acknowledge that the limited number of studies and relatively small participant samples included in the meta‐analysis may have influenced the statistical power of these comparisons. Additional research encompassing larger cohorts is necessary to further clarify the comparative efficacy of HIIT and LIET interventions in youth athlete populations.

### 4.2 HIIT versus generic training

When compared to GT, HIIT demonstrated significant advantages in improving VO_2_max among youth athletes (Hedges' *g* = 0.97; 95% CI: 0.65 to 1.28; *p* < 0.01). This substantial effect size indicates that HIIT is highly effective in enhancing aerobic capacity compared to GT, which often consists of general endurance activities performed at moderate intensities, technical drills, and non‐specific conditioning exercises.

Initially, HIIT did not significantly differ from GT in improving endurance performance measures (Hedges' *g* = 0.59; 95% CI: −0.19 to 1.37; *p* = 0.14). However, after conducting a sensitivity analysis and excluding an outlier study (Breil et al., 2010), HIIT significantly improved endurance performance more than GT (Hedges' *g* = 0.91; 95% CI: 0.42 to 1.40; *p* < 0.01). The outlier study reported methodological concerns that might have negatively influenced performance in the time‐to‐exhaustion test due to testing protocols (short rest between VO_2_max test and time to exhaustion test), potentially explaining its divergence from other studies. The exclusion of the outlier did not change the level of significance of the results.

The superior effectiveness of HIIT over GT may be attributed to the higher intensity and specific overload provided by HIIT protocols. HIIT involves exercising at near‐maximal intensities, stimulating significant cardiovascular and metabolic adaptations essential for endurance performance (Buchheit & Laursen, 2013b). In contrast, GT may not consistently provide the necessary intensity or specificity to elicit maximal improvements in aerobic capacity. However, for an evidence‐informed statement, more studies are needed that implement direct comparisons of intensity parameters between groups. The studies included in the current analysis mostly lack comparable intensity information (Table 4).

Moreover, HIIT allows for precise manipulation of training variables such as intensity, duration, and recovery intervals, enabling coaches to target specific physiological adaptations (Iaia & Bangsbo, 2010). This level of control is often lacking in GT programs, which may include a broader range of activities with varying intensities and objectives.

### 4.3 HIIT versus sport‐specific drills

The comparison between HIIT and SSD showed that both training modalities are effective in improving VO_2_max, with no significant difference observed (Hedges' *g* = 0.13; 95% CI: −0.16 to 0.42; *p* = 0.38). This suggests that SSD can be as effective as HIIT in enhancing aerobic capacity. SSD is designed to replicate the physiological demands of actual match play by incorporating sport‐specific movements and decision‐making within a game context (Clemente et al., 2014). The high‐intensity intermittent efforts required during SSD closely mimic those in HIIT, leading to similar cardiovascular and metabolic responses (Hill‐Haas et al., 2011).

However, HIIT demonstrated a significant advantage over SSD in improving endurance performance measures (Hedges' *g* = 0.41; 95% CI: 0.07 to 0.75; *p* = 0.02). This indicates that while both modalities can enhance VO_2_max, HIIT may be more effective in improving specific endurance performance outcomes such as time trials or maximal aerobic speed. The structured and controlled nature of HIIT allows for precise targeting of training intensities and durations, potentially leading to greater improvements in endurance performance (Seiler & Tønnessen, 2009).

No significant differences were found between HIIT and SSD in intermittent endurance performance and RSA, suggesting that both modalities are equally effective in developing these specific fitness components. This may be due to the similar intermittent high‐intensity demands of both training methods, which stimulate adaptations in anaerobic capacity and neuromuscular function (Kunz et al., 2019).

The sport‐specific nature of SSD also allows for simultaneous development of technical and tactical skills, which is an added advantage over HIIT (Clemente et al., 2014). Coaches may prefer SSD when the goal is to enhance both physical fitness and sport‐specific skills, whereas HIIT may be more suitable when the primary focus is on improving endurance performance.

### 4.4 Strengths and limitations

This meta‐analysis offers several notable strengths that enhance the clarity and applicability of its findings. By explicitly comparing HIIT with LIET, GT, and SSG, the present study extends beyond conventional research approaches, filling a critical gap in the literature and providing a more nuanced understanding of the relative efficacy of these training modalities. Furthermore, a structured hierarchical approach ensured that only the most relevant and comparable outcome measures were analyzed, mitigating the potential for biased interpretations and enhancing the precision of our conclusions. In addition, the use of sensitivity analyses, including outlier and influence diagnostics, bolstered the robustness of the results, ensuring that no single study with methodological limitations unduly influenced the overall findings.

Despite these strengths, several limitations must be acknowledged. Firstly, significant heterogeneity existed among the included studies regarding participant age, sport type, training protocols, and outcome measures. While random‐effects models were used to account for variability, residual heterogeneity may still have influenced the results, potentially affecting the robustness of the conclusions. Secondly, the limited female representation restricts the generalizability of the findings to female youth athletes, as only 10.7% of participants were female. Future studies should aim for a more balanced gender representation to enhance applicability across sexes. Thirdly, some comparisons involved limited sample sizes, notably in the HIIT versus LIET comparison, which may reduce statistical power and affect generalizability. Additionally, due to the small number of studies, no distinctions could be made regarding different HIIT protocols. It is possible that specific protocols like sprint interval training may elicit different neuromuscular adaptations compared to long‐interval HIIT in certain performance aspects. Furthermore, there were too few studies analyzing different methods of incorporating GT with HIIT—such as adding HIIT to GT, replacing parts of GT with HIIT, or varying interventions between groups—making it unfeasible to conduct more in‐depth analyses on these approaches. The possibility of publication bias cannot be entirely excluded. Although funnel plots appeared symmetrical, the small number of studies limits the ability to conclusively rule out publication bias, which could affect the validity of the results. Additionally, most included studies focused on short‐term interventions ranging from 4 to 10 weeks.

Consequently, the long‐term sustainability of the observed performance improvements remains unclear, necessitating longer‐duration studies to determine if benefits persist over time. Although some funnel plots displayed studies outside the pseudo 95% confidence boundaries, this is not uncommon in meta‐analyses with small sample sizes and does not necessarily indicate publication bias. Nonetheless, the possibility of selective reporting cannot be entirely excluded.

While we aimed to include only youth athletes, the operational definition of ‘athlete’ in childhood and adolescence remains a challenge due to considerable variability in biological maturation and training exposure. We pragmatically defined youth athletes as individuals engaged in regular, structured sport‐specific training. However, no standardized performance‐based criteria were applied. Furthermore, only healthy youth athletes were included, defined by the absence of chronic or acute medical conditions. Importantly, only studies including children and adolescents aged 13 years and older were analyzed; thus, no information is available on the effects of these training modalities in younger athletes. Future research may benefit from including more detailed descriptions of training history, maturity status, health conditions, and sport specialization to allow for more precise subgroup analyses.

Training season and contextual factors such as environmental temperature or competitive phase may have influenced responses but were rarely reported and are thus a source of unexplained variance. Another important limitation is the lack of information on biological maturation across the included studies. Since children and adolescents exhibit considerable interindividual variability in maturity status, particularly during puberty, this can substantially affect training responsiveness and performance outcomes. Unfortunately, very few studies reported markers of maturation (e.g., Tanner stage or years from peak height velocity), making it impossible to account for this variable in our analyses. For improved comparability and interpretability, future research should aim to include standardized assessments of biological maturation when studying exercise effects in youth populations.

Although only randomized controlled trials were included, variation in study quality, sample sizes, and intervention characteristics may have influenced effect estimates and should be considered when interpreting the results.

### 4.5 Practical applications

Coaches and practitioners seeking to develop aerobic and anaerobic endurance performance in youth athletes can enhance the training effectiveness by integrating HIIT into their programs. The current results suggest that prioritizing HIIT is advisable when the goal is to improve aerobic capacity. The structured nature of HIIT allows for precise control over intensity and work‐to‐rest ratios, enabling coaches to target specific physiological adaptations crucial for endurance performance.

In addition, SSD and HIIT can offer synergistic benefits. While HIIT provides targeted physiological stimuli, SSD incorporates technical and tactical skill development within an enjoyable and interactive format. This combination can enhance motivation and adherence among youth athletes—critical factors for long‐term engagement in sports (Katis & Kellis, 2009). The enjoyable nature of SSD can make training sessions more appealing, thereby encouraging consistent participation and effort.

When designing training programs, coaches should consider the individual needs, preferences, and developmental stages of their athletes. Balancing the structured demands of HIIT with the variety and enjoyment offered by SSD can cater to different learning styles and keep athletes engaged. Tailoring training plans in this way not only optimizes physiological adaptations but also supports the overall development of the athlete.

## 5 CONCLUSION

This meta‐analysis underscores the efficacy of HIIT as a potent training modality for enhancing aerobic capacity and endurance performance in youth athletes. HIIT outperforms generic training and shows equal effectiveness as low‐intensity endurance training in improving endurance performance and VO_2_max, offering a time‐efficient and effective approach. While HIIT and SSD are similarly effective in enhancing VO_2_max, HIIT provides greater improvements in endurance performance measures. Coaches should consider incorporating HIIT into training regimens to maximize physiological adaptations relevant to endurance performance. A combined approach that integrates HIIT and sport‐specific games may offer comprehensive benefits, addressing various physiological, technical, and psychological aspects of athlete development.

Given the relatively small number of studies included per outcome (ranging from 3 to 9), the reported effects should be interpreted with caution—particularly as most of the analyzed parameters (e.g., VO_2_max, RSA, TTE) are methodologically straightforward to assess in youth sport settings. Future research should focus on long‐term studies with larger and more diverse samples, including a greater proportion of female athletes, to further elucidate the effects of these training modalities across different age groups and sports.

## Supporting information


**Data S1.** Supporting Information.
