# Supplementary material for: Meta‐analysis of high‐intensity interval training and alternative modalities for enhancing aerobic and anaerobic endurance in young athletes
Source: Physiol Rep. 2025 Oct 5;13(19):e70598. doi: 10.14814/phy2.70598 (PMC12497894; doi:10.14814/phy2.70598)
Supplement: Supplementary file 1 — Data S1. Supporting Information. [file PHY2-13-e70598-s001.pdf]

Funnel Plot: HIIT versus LIET - variables of sustained aerobic performance

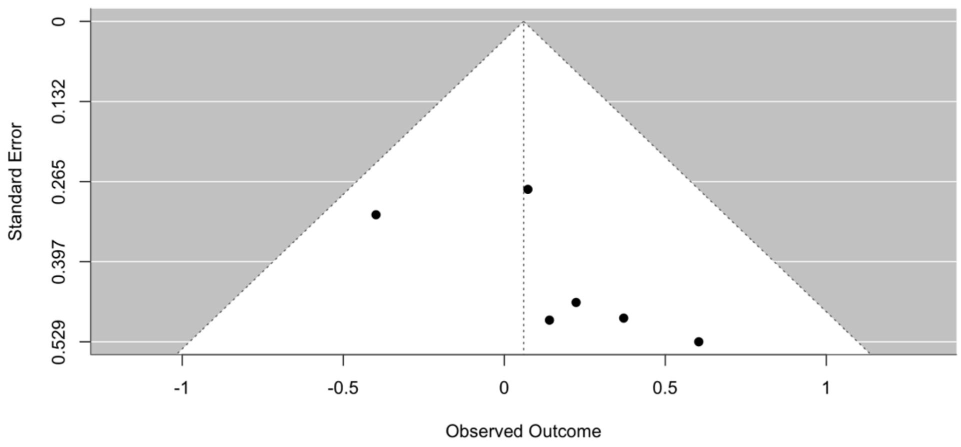

Funnel Plot: HIIT versus LIET - variables of sustained aerobic performance (after sensitivity analysis)

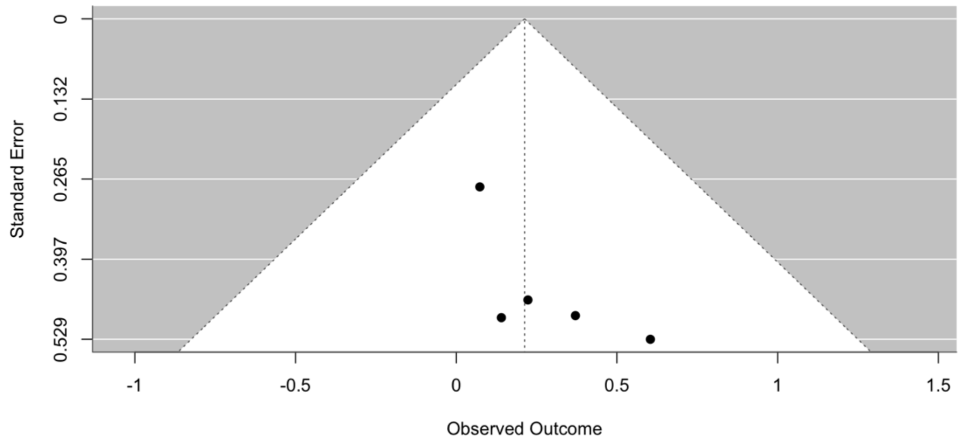

Funnel Plot: HIIT versus LIET – VO_{2max}

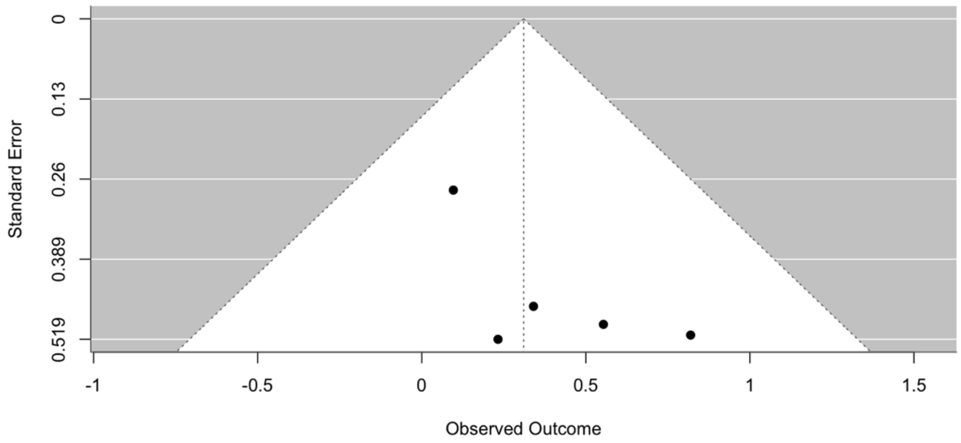

Funnel Plot: HIIT versus GT – VO_{2max}

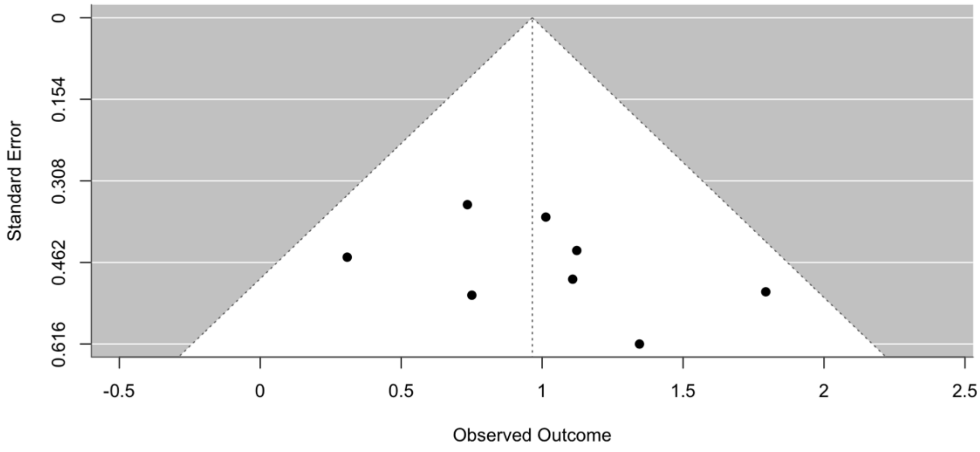

Funnel Plot: HIIT versus GT - variables of sustained aerobic performance

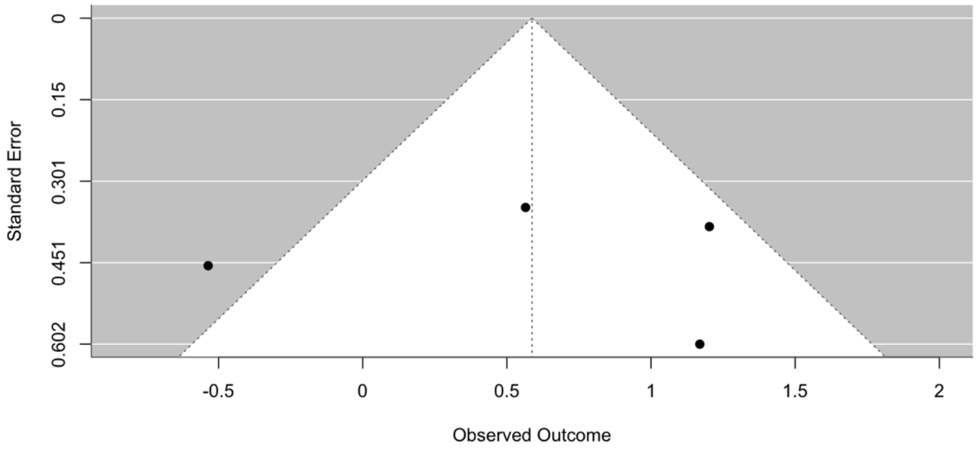

Funnel Plot: HIIT versus GT - variables of sustained aerobic performance (after sensitivity analysis)

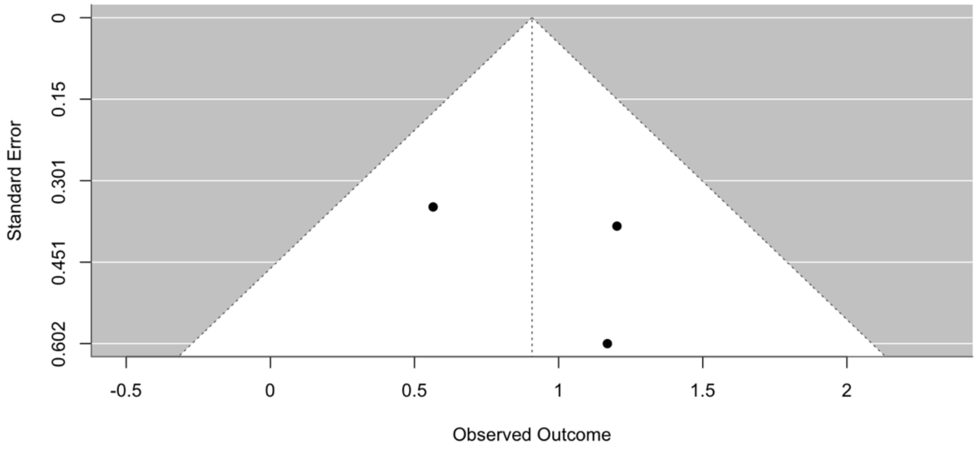

Funnel Plot: HIIT versus SSD – VO_{2max}

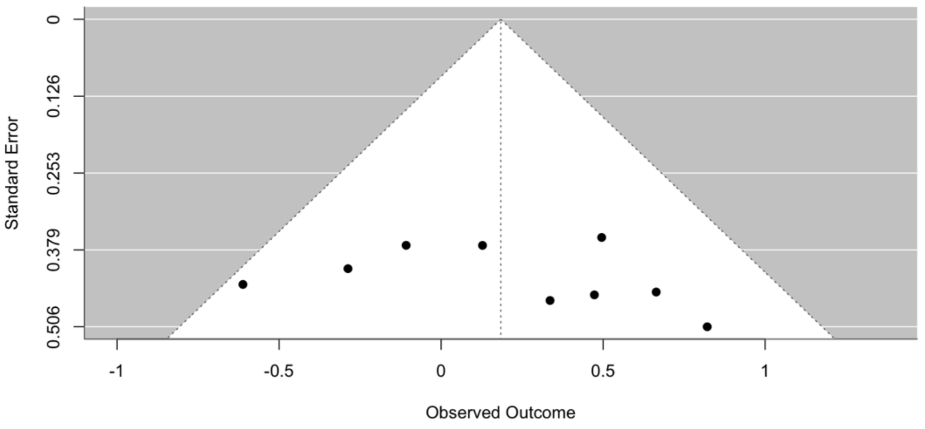

Funnel Plot: HIIT versus SSD - variables of sustained aerobic performance

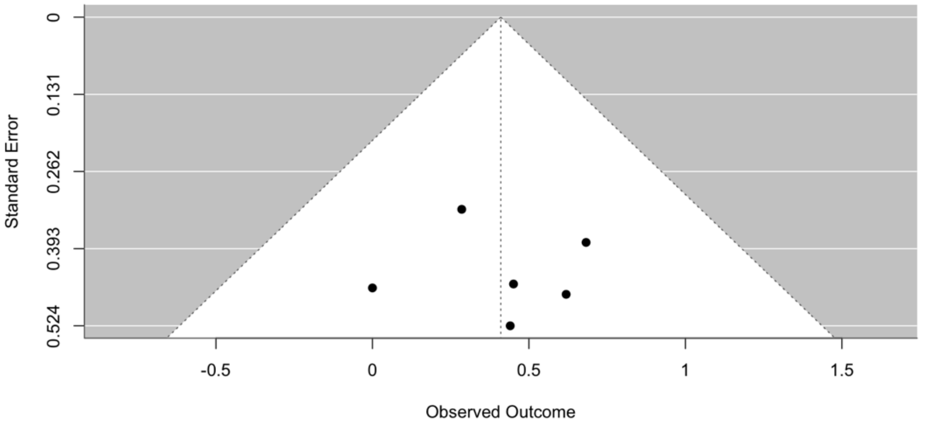

Funnel Plot: HIIT versus SSD - variables of intermittent endurance performance

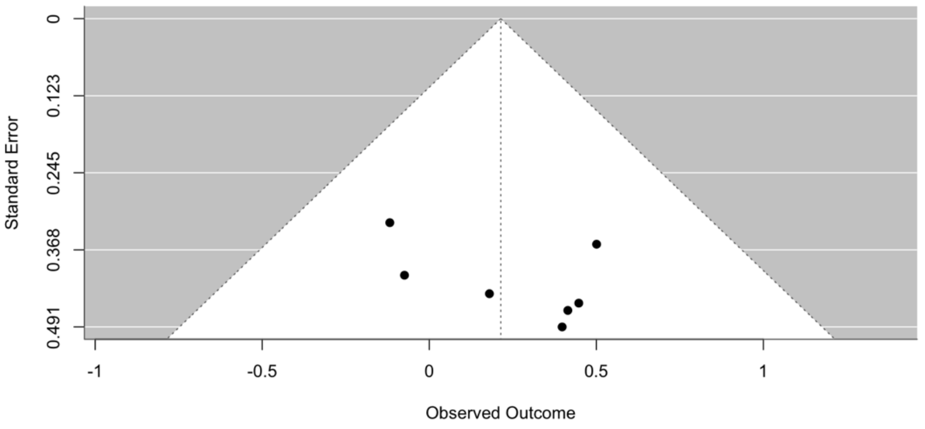

Funnel Plot: HIIT versus SSD – Repeated Sprint Ability

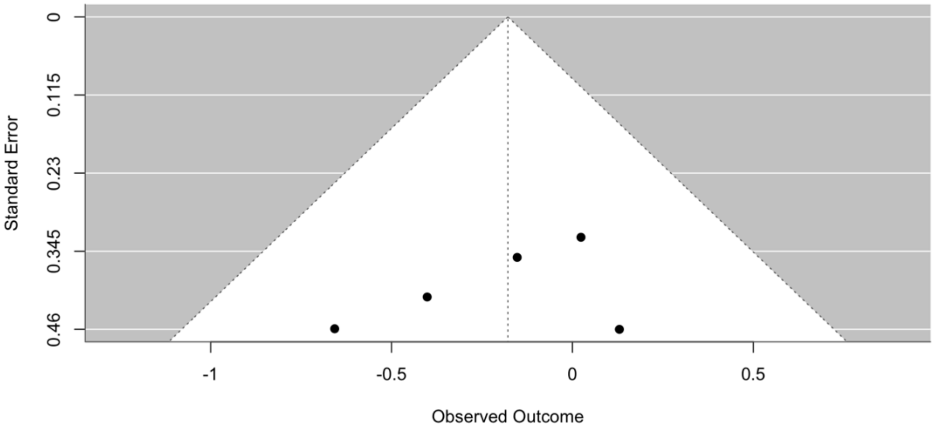

HIIT vs LIET - variables of sustained aerobic performance

Author (Year)	Sport	Test	n1i	n2i	Training Modality	m_pre_1i	sd_pre_1i	m_post_1i	sd_post_1i	m_pre_2i	sd_pre_2i	m_post_2i	sd_post_2i	mean_change_1i	mean_change_2i	sd_pooled	Morris' adjustm	yi	vi	sei	rb.a	rb.b	rb.c	rb.d	rb.e	rb.f
Faude et al. (2013)	Soccer	IAT	20	20	Running	13.25	0.63	13.71	0.91	13.3	0.96	14.09	0.89	0.46	0.79	0.81	0.98	-0.40	0.10	0.32	+	?	+	+	+	+
Sperlich et al. (2010)	Swimming	TT	26	26	Swimming	2158	304	2097	272	2145	314	2107	277	-61	-38	309.04	0.98	0.07	0.08	0.28	+	?	+	+	+	+
Ketelhut et al. (2020)	Rowing	TTE	10	7	Rowing	923	222	981	226	829	225	854	204	58	25	223.20	0.95	0.14	0.24	0.49	+	?	+	+	+	+
Sandbakk et al. (2013)	XC Ski	TT	14	7	Roller Ski	750.5	196.6739	688	185.436	705	194	688	164	-62.5	-17	195.83	0.96	0.22	0.22	0.46	+	?	+	+	+	+
Sperlich et al. (2011)	Soccer	TT	9	8	Running	234	12.8	224	13.8	235	12.8	230	17.3	-10	-5	12.80	0.95	0.37	0.24	0.49	+	?	+	+	+	+
Sandbakk et al. (2011)	XC Ski	TT	7	8	Roller Ski	222	23	212	20	211	14	213	11	-10	2	18.70	0.94	0.60	0.28	0.53	+	?	+	+	+	+

RED = Excluded after sensitivy analysis.

HIIT vs LIET - VO2max

Author (Year)	Sport	n1i	n2i	Training Modality	m_pre_1i	sd_pre_1i	m_post_1i	sd_post_1i	m_pre_2i	sd_pre_2i	m_post_2i	sd_post_2i	mean_change_1i	mean_change_2i	sd_pooled	Morris' adjustm	yi	vi	sei	rb.a	rb.b	rb.c	rb.d	rb.e	rb.f
Sperlich et al. (2010)	Swimming	26	26	Swimming	39.9	8.9	44.5	7.2	39.4	9.5	43.1	6.7	4.6	3.7	9.20	0.98	0.10	0.08	0.28	+	?	+	+	+	+
Sandbakk et al. (2011)	XC Ski	7	8	Roller Ski	67.5	6.5	70.2	6.8	69.3	7.2	70.3	7.3	2.7	1	6.89	0.94	0.23	0.27	0.52	+	?	+	+	+	+
Sandbakk et al. (2013)	XC Ski	14	7	Roller Ski	67	7.071068	69.5	7.091662	68	7	68	7	2.5	0	7.05	0.96	0.34	0.22	0.47	+	?	+	+	+	+
Sperlich et al. (2011)	Soccer	9	8	Running	55.1	4.9	58.9	4.7	55.3	4.3	56.4	3.7	3.8	1.1	4.63	0.95	0.55	0.25	0.50	+	?	+	+	+	+
Ketelhut et al. (2020)	Rowing	10	7	Rowing	58.4	3.75	62.1	3.67	58.4	5.23	58.3	5.95	3.7	-0.1	4.40	0.95	0.82	0.26	0.51	+	?	+	+	+	+

HIIT vs GT - VO2max

Author (Year)	Sport	n1i	n2i	Training Modality	m_pre_1i	sd_pre_1i	m_post_1i	sd_post_1i	m_pre_2i	sd_pre_2i	m_post_2i	sd_post_2i	mean_change_1i	mean_change_2i	sd_pooled	Morris' adjustm	yi	vi	sei	rb.a	rb.b	rb.c	rb.d	rb.e	rb.f
Breil et al. (2010)	Alpin Skiing	13	8	cycling & obstacle running	53	4.6	56.2	5.1	52.9	6.3	54.4	7	3.2	1.5	5.29	0.96	0.31	0.20	0.45	?	?	+	+	+	?
Seo et al. (2019)	Teakwondo	36	11	running	64.43	6.15	68.83	7.25	63	5.54	62.9	6.53	4.4	-0.1	6.02	0.98	0.73	0.12	0.35	+	?	+	+	+	+
Sommer Jeppesen et al. (2022)	Ice Hockey	11	6	ice skating	51.71	3.37	54.39	4.35	51.71	5.75	50.98	6.3	2.68	-0.73	4.31	0.95	0.75	0.27	0.52	+	?	+	+	+	+
Hammami et al. (2021)	Handball	17	15	sprint + plyo	47.8	2.5	52.2	1.9	48.4	2.9	50	2.6	4.4	1.6	2.69	0.97	1.01	0.14	0.38	+	?	+	+	+	+
Helgerud et al. (2001)	Soccer	9	10	running	58.1	4.5	64.3	3.9	58.4	4.3	59.5	4.4	6.2	1.1	4.40	0.96	1.11	0.24	0.49	+	?	+	+	+	+
Ouergui et al. (2020)	Teakwondo	12	12	running	39.6	3.6	44.2	3.9	37.2	4.9	36.8	5.4	4.6	-0.4	4.30	0.97	1.12	0.19	0.44	+	?	+	+	+	+
Thom et al. (2019)	Soccer	7	6	cycling	54.23	5.17	59.42	5.48	58.27	7.21	54.52	11.54	5.19	-3.75	6.18	0.93	1.35	0.38	0.62	+	?	+	+	+	+
Impellizzeri et al. (2008)	Soccer	11	10	Running	56.6	2	58.9	3	57.7	1	57	1	2.3	-0.7	1.61	0.96	1.79	0.27	0.52	+	?	+	+	+	+

HIIT vs GT - variables of sustained aerobic performance

Author (Year)	Sport	Test	n1i	n2i	Training Modality	m_pre_1i	sd_pre_1i	m_post_1i	sd_post_1i	m_pre_2i	sd_pre_2i	m_post_2i	sd_post_2i
Breil et al. (2010)	Alpin Skiing	Tlim	13	8	cycling & obstacle running	137	20	144	24	148	28	168	41
Seo et al. (2019)	Teakwondo	TTE	36	11	running	15.77	1.27	16.27	1.27	15.8	1.03	15.6	1.5
Thom et al. (2019)	Soccer	TTE	7	6	cycling	654	54	691	53	598	61	563	84
Hammami et al. (2021)	Handball	MAS	17	15	sprint + plyo	14.8	0.6	15.9	0.5	15	0.7	15.3	0.6

Author (Year)	mean_change_1i	mean_change_2i	sd_pooled	Morris' adjustm	yi	vi	sei	rb.a	rb.b	rb.c	rb.d	rb.e	rb.f						
Breil et al. (2010)	7	20	23.27	0.96	-0.54	0.21	0.46	+	?	?	+	+	+	?	?	+	+	+	?
Seo et al. (2019)	0.5	-0.2	1.22	0.98	0.56	0.12	0.35	+	?	?	+	+	+	+	?	+	+	+	+
Thom et al. (2019)	37	-35	57.29	0.93	1.17	0.36	0.60	+	?	?	+	+	+	+	?	+	+	+	+
Hammami et al. (2021)	1.1	0.3	0.65	0.97	1.20	0.15	0.38	+	?	?	+	+	+	+	?	+	+	+	+

RED = Excluded after sensitivy analysis.

HIIT vs SSD - VO2max

Author (Year)	Sport	n1i	n2i	Training Modality	m_pre_1i	sd_pre_1i	m_post_1i	sd_post_1i	m_pre_2i	sd_pre_2i	m_post_2i	sd_post_2i	mean_change_1i	mean_change_2i	sd_pooled	Morris' adjustm	yi	vi	sei	rb.a	rb.b	rb.c	rb.d	rb.e	rb.f
Jastrzebski et al. (2014)	Soccer	11	11	running	55.7	5.23	56.9	5.58	52.5	5.15	57	5.44	1.2	4.5	5.19	0.96	-0.61	0.19	0.44	+	?	+	+	+	+
Ouergui et al. (2020)	Teakwondo	12	12	running	39.6	3.6	44.2	3.9	39.8	3.1	45.4	4.2	4.6	5.6	3.36	0.97	-0.29	0.17	0.41	+	?	+	+	+	+
Kilit & Arslan (2018)	Tennis	14	15	running	45.4	1.9	47.9	1.8	45.9	1.7	48.6	1.9	2.5	2.7	1.80	0.97	-0.11	0.14	0.37	+	?	?	+	+	+
Impellizzeri et al. (2006)	Soccer	15	14	running	55.6	3.4	60.2	3.9	57.7	4.2	61.8	4.5	4.6	4.1	3.81	0.97	0.13	0.14	0.37	+	?	+	+	+	+
Hill-Haas et al. (2009)	Soccer	9	10	running	60.2	4.6	61.4	3.5	59.3	4.5	58.9	5.5	1.2	-0.4	4.55	0.96	0.34	0.21	0.46	+	?	+	+	+	+
Arslan et al. (2020)	Soccer	10	10	running	46.8	0.6	48.9	0.9	47.2	1.3	48.8	0.8	2.1	1.6	1.01	0.96	0.47	0.21	0.45	+	?	+	+	+	?
Arslan et al. (2022)	Basketball	16	16	running	45.89	0.85	48.18	0.67	46.29	1.03	48.1	0.87	2.29	1.81	0.94	0.97	0.50	0.13	0.36	+	?	+	+	+	?
Harrison et al. (2015)	Field hockey & Rugby	11	10	running	55.9	2.5	59	2.3	55.9	3	57.1	3.5	3.1	1.2	2.75	0.96	0.66	0.20	0.45	+	?	+	+	+	+
Fernandez-Fernandez et al. (2016)	Tennis	8	9	running	56.2	3.1	59.7	3.3	56.1	2.2	57.3	2.1	3.5	1.2	2.66	0.95	0.82	0.26	0.51	+	?	+	+	+	+

HIIT vs SSD - variables of sustained aerobic performance

Author (Year)	Sport	Test	n1i	n2i	Training Modality	m_pre_1i	sd_pre_1i	m_post_1i	sd_post_1i	m_pre_2i	sd_pre_2i	m_post_2i	sd_post_2i	mean_change_1i	mean_change_2i	sd_pooled	Morris' adjustm	yi	vi	sei	rb.a	rb.b	rb.c	rb.d	rb.e	rb.f
Faude et al. (2014)	Soccer	IAT	9	10	running	14.3	0.9	14.5	0.7	14.3	0.8	14.5	0.7	0.2	0.2	0.85	0.96	0.00	0.21	0.46	+	?	+	+	+	+
Buchheit et al. (2009)	Handball	Tlim (IFT)	19	19	running	180	96	245	115	155	66	196	102	65	41	82.38	0.98	0.29	0.11	0.33	+	?	+	+	+	+
Los Arcos et al. (2015)	Soccer	MAS	8	7	running	16.8	0.9	17.1	1	17	0.8	16.9	0.8	0.3	-0.1	0.86	0.94	0.44	0.27	0.52	+	?	+	+	+	+
Arslan et al. (2020)	Soccer	TT	10	10	running	243	17	229	14	236	17	230	17	-14	-6	17.00	0.96	0.45	0.21	0.45	+	?	+	+	+	?
Hill-Haas et al. (2009)	Soccer	TTE	9	10	running	589	34	604	43	594	50	581	49	15	-13	43.21	0.96	0.62	0.22	0.47	+	?	+	+	+	+
Kilit & Arslan (2018)	Tennis	TT	14	15	running	74.9	2.6	71.4	2.7	75.4	2.8	73.8	2.5	-3.5	-1.6	2.71	0.97	0.68	0.15	0.38	+	?	?	+	+	+

<u>HIIT vs SSD - variables of intermittent endurance performance</u>

Author (Year)	Sport	Test	n1i	n2i	Training Modality	m_pre_1i	sd_pre_1i	m_post_1i	sd_post_1i	m_pre_2i	sd_pre_2i	m_post_2i	sd_post_2i	mean_change_1i	mean_change_2i	sd_pooled	Morris' adjustm	yi	vi	sei	rb.a	rb.b	rb.c	rb.d	rb.e	rb.f
Buchheit et al. (2009)	Handball	VIFT	19	19	running	17.9	1.8	18.9	1.3	18.4	1.5	19.6	1.4	1	1.2	1.66	0.98	-0.12	0.11	0.32	+	?	+	+	+	+
Delextrat & Martinez (2014)	Basketball	VIFT	12	12	running	17.4	0.7	18	1	17.2	1.7	17.9	1.5	0.6	0.7	1.30	0.97	-0.07	0.17	0.41	+	?	+	+	+	+
Harrison et al. (2015)	Field hockey & Rugby	VIFT	11	10	running	18.4	0.8	19.6	0.7	18.1	1.3	19.1	1.4	1.2	1	1.07	0.96	0.18	0.19	0.44	+	?	+	+	+	+
Fernandez-Fernandez et al. (2016)	Tennis	VIFT	8	9	running	18	0.9	18.8	0.8	17.9	1	18.3	0	0.8	0.4	0.95	0.95	0.40	0.24	0.49	+	?	+	+	+	+
Hill-Haas et al. (2009)	Soccer	YYIRT-1	9	10	running	1764	256	2151	261	1488	345	1742	362	387	254	306.36	0.96	0.41	0.22	0.46	+	?	+	+	+	+
Arslan et al. (2020)	Soccer	YYIRT-1	10	10	running	1240	75	1484	74	1284	152	1472	99	244	188	119.85	0.96	0.45	0.21	0.45	+	?	+	+	+	?
Arslan et al. (2022)	Basketball	YYIRT-1	16	16	running	1130	100.66	1402.5	79.29	1177.5	122.17	1392.5	103.51	272.5	215	111.93	0.97	0.50	0.13	0.36	+	?	+	+	+	?

HIIT vs SSD - RSA

Author (Year)	Sport	Test	n1i	n2i	Training Modality	m_pre_1i	sd_pre_1i	m_post_1i	sd_post_1i	m_pre_2i	sd_pre_2i	m_post_2i	sd_post_2i	mean_change_1i	mean_change_2i	sd_pooled	Morris' adjustm	yi	vi	sei	rb.a	rb.b	rb.c	rb.d	rb.e	rb.f
Arslan et al. (2020)	Soccer	RSA	10	10	running	38.2	1.7	34.9	1.5	37.8	1.5	35.6	1.2	-3.3	-2.2	1.60	0.96	-0.66	0.21	0.46	+	?	+	+	+	?
Delextrat & Martinez (2014)	Basketball	RSA	12	12	running	27.1	1.9	27	1.8	27.9	2.4	28.7	1.9	-0.1	0.8	2.16	0.97	-0.40	0.17	0.41	+	?	+	+	+	+
Arslan et al. (2022)	Basketball	RSA	16	16	running	36.66	0.83	34.65	0.79	36.87	1.29	35.03	1.3	-2.01	-1.84	1.08	0.97	-0.15	0.13	0.35	+	?	+	+	+	?
Buchheit et al. (2009)	Handball	RSA	19	19	running	6.27	0.42	6.05	0.37	6.19	0.41	5.96	0.31	-0.22	-0.23	0.42	0.98	0.02	0.11	0.32	+	?	+	+	+	+
Hill-Haas et al. (2009)	Soccer	RSA	9	10	running	42.2	1.8	42.3	1.5	42.1	1.1	42	1.4	0.1	-0.1	1.47	0.96	0.13	0.21	0.46	+	?	+	+	+	+
